# Obstinate Epilepsy, Treatment

**Published:** 1874-02

**Authors:** Allan McLane Hamilton


### Our Exchanges.

---

*THE MEDICAL RECORD—December*, 1873:

TREATMENT OF OBSTINATE EPILEPSY—*By Allan McLane Hamilton, M.D.*—The want of permanence in the cure of those cases of epilepsy (by bromides especially) when there is a relapse, after the patient and physician have congratulated themselves upon the mastery of disease, is, I am convinced, due to an insufficient use of these drugs.

The pathological condition of the brain and its appendages in old cases, may be such as to make the cure impossible, or almost so. My purpose was to discuss the management of those hopeless cases that the practitioner meets at the dispensaries, or who come to him after years of suffering, and after running the gauntlet of drugs, from cotyledon umbilicus to nitrate of silver. Some of them may have been taking the bromides, or equally efficacious remedies, in an irregular way without benefit, and have given up, disgusted and despondent. Conium and hyoscyamus have been used by some practitioners, but the bromides and two or three others have been so successful that they are now generally adopted. My experience has taught me to rely upon a few good remedies. Ergot and belladonna are the best agents I know of as local alteratives, while quinine and cod-liver oil will help the general condition. I propose to dwell briefly upon three drugs which I am convinced give the best results, and these are: the bromides f sodium and potassium, belladonna, and ergot.

Before their administration, however, we must inquire as regards the condition of the patient, his age, duration of sickness, and the existence of a proximate or remote cause; and we should discover whether the attacks be diurnal or nocturnal

The bromides are almost useless in nocturnal epilepsy, and they often aggravate the symptoms, but are invaluable when the attacks come on in the day-time. The bromides produce slower effects in those cases of long standing than they do in recent ones. In cases where *le petit mal* is the feature, belladonna and ergot are particularly indicated, and not the bromide, which increases the severity and number of the seizures.

The bromides, according to Clarke and Amory, produce a contraction of the arterioles of the medulla, and this contraction must be permanent to prevent a recurrence of the paroxysm. Dr. Russel Reynolds says that a considerable amount of these salts should be present in the system, and that is uot obtained by the mere administration of small repeated doses. This statement substantiates my assertion, that in my experience those patients who took heroic doses were cured soonest. I believe that a state closely bordering on bromism is to be reached before we can expect permanency of cure. We must stop here, however, and employ the greatest caution in their subsequent use. Staggering, anæsthesia of the fauces, and mental cloudiness are to be reached; after these come a train of deplorable symptoms which it is almost impossible to stop. The skin will be characterized by a waxy hue; œdema of the limbs and loose tissue beneath the lower eyelids; utter loss of appetite (not always constant), and a mental state of imbecility will ensue. The patient will fall into a low state of exhaustion, attended by delirium, irritable pulse, coldness of skin, followed by a sudden rise of temperature, and finally death.

I have usually begun with twenty grains of the bromide of sodium thrice daily. At the end of a week, if there is no change for the better or worse, the dose may be increased by ten grains, and so on till we give one hundred and sixty grains in the course of the day. It is then necessary to watch carefully for the appearance of symptoms which will indicate the toxic effects of the drug. The patient may be seized with an irresistible desire to sleep at all times. He may fall asleep over the table, or may appear absent-minded. If spoken to, his mind will sometimes be "wool-gathering," and he will not heed the first summons, but regain himself suddenly to pay attention, which is an effort. As other symptoms increase, he will display an irritability of temper, an indisposition to go out into the open air, a staggering gait combined with a bending of the knees, and consequent sinking of the body. His face will wear a blank, expressionless appearance, and the color of his skin will appear a dirty white. His breath becomes foul with the odor of bromine, and his pharynx devoid of sensibility. An eruption of acne may be present on the forehead and chin.

As we advance into the second stage we find that a number of alarming symptoms will develop themselves in succession. The process of waste goes on with excessive rapidity; a loose cough and an œdematous condition of the lung-tissue will ensue; in fact, there may be anasarca of the entire body when the patient is bromized to this extent. As the stomach has been a sufferer for a long time, it is reasonable to expect a change in this organ. The secretion of gastric juice is interferred with, and it is often extremely difficult to find an article of diet that will be digested. The patient's pulse becomes weak and intermitting, large dark circles surround the eyes, followed by total mental aberration, and and then come the signs of dissolution alluded to before. The symptoms towards the close of life may resemble those of typhoid.

After reading this brief list of symptoms, we have certainly cause to avoid its excessive use; but if care is used the patient may be kept hovering on the borders of bromism without going further. Certain adjuvants in the treatment will stave off the severe effects of the bromides, and when bromism is produced will quickly restore the patient. The various preparations of cinchona and iron, combined perhaps with cod-liver oil or strychnia in small doses, will do almost immediate good.

It has been suggested that the combination of strychnine with the bromides is efficacious; but the effect of this alkaloid, in the cases I have seen and treated, has been to aggravate the disease.

*Belladonna*, given by the stomach *with the bromides*, has also been tried; but the properties of atropia are apt to be neutralized by the alkaline bases of these salts, particularly calcium.

My experience has led me to believe that the variety of epileptic seizures occurring in the day-time were most readily abated by this form of treatment. Large doses of belladonna greatly lessened the form of attack known as *le petit mal.* Belladonna, as we know, diminishes the amount of blood in the vessels of the cord, and establishes a condition antagonistic to spasm of these vessels. Trousseau's method of giving belladonna, which I have taken from Ringer, is this: "During the first month of treatment the patient takes a pill composed of ext. of belladonna and the powdered leaves of belladonna, of each one-fifth part of a grain, every day, if his attacks occur chiefly in the day-time, or in the evening, if they are nocturnal. One pill added to the dose every month; and whatever be the dose, it is always taken at the same period of the day. By this means the patient may reach the dose of from five to twenty pills, and even more."

As yet I have not heard of the administration of belladonna and its alkaloid by the hypodermic method, locally. In several cases I have made this the sole treatment, and though it was tried somewhat empirically, I had the gratification of witnessing most important results. From one-hundredth, increased to one-forty-eighth of a grain of the sulphate of atropia solution, were injected beneath the skin at the base of the skull, for several weeks, with intervals of one or more days between each application. The want of sensibility of this part of the skin made the pain inconsiderable, and the patient scarcely minded the operation, repeated even as often as it was. The effect seemed to be more immediate than when belladonna was given by the mouth. The results were gratifying, inasmuch as one case of ten years' standing was improved notably after three weeks; and another case, when this treatment was combined with the bromide, was cured.

*Ergot.*—My experiments have been made with the ergotine of Bonjean of Paris, and propylamin, which is indentical with secaline, the active principle of ergot. I have several cases under my care which have notably improved under the ergotine treatment. Those cases the most benefited were of the variety known as *la petit mal.* One of Bonjean's capsules, containing about six grains given three times daily, is enough to begin with. The fluid extract produced less satisfactory results. Propylamin has been tried in one case in doses of ♏v. suspended in mucilage. Though this case is unusually severe and has been under all kinds of treatment, a great diminution in the number of attacks is evident.

The influence of ergot on the muscular coat of the blood-vessels of the cord is to contract them, and restore their tone through the vaso-motor nerves. Its action is much the same as that of belladonna, though in a far more energetic degree. Ergot notably diminishes exaggerated reflex power, and is therefore clearly indicated in epilepsy. It will combine well with the bromides, and the therapeutical effects of both will be increased.

In spite of the medicines given, the physician will sometimes find that his patient does not recover, and perhaps he will give the case up, feeling that nothing can be done. We should always look for a deeper evil in these cases, for a hidden cause may exist in very many instances, which, when removed, there may be a rapid cure.

Uterine and ovarian derangements are a very common cause of the disorder. The prospect of cure depends upon the return of function through disappearance of the cause, and consequent return of tone of the cerebro-spinal system. The hope of benefit in these cases depends upon the time of life, and the proximity to the commencement of menstruation. It is much easier to cure a young patient than one in whom the disease has become deep-seated. Any prolonged irritation of the pudic nerve is apt, whether in the male or female, to be followed by epilepsy, and such causes of irritation it is well to discover.

Besides these causes, we will be able to find that injuries of the head, ascarides, and renal and biliary calculi may also be frequent causes of the trouble. A form of epilepsy which gives the physician much annoyance, is that depending upon or caused by disturbances of the digestive apparatus, and this occurs in persons having a nervous diathesis. The important part which the stomach plays in epilepsy may be studied when we watch the disagreeable results which follow an abuse of this organ.

There are other kinds of eccentric irritation which will cause epilepsy, and may escape the attention of the practitioner; a carious tooth, or an injury of the jaw; the presence of some irritating body such as a needle or bullet.

Those forms of epilepsy which depend upon injuries of the head or intracranial tumors resist all forms of treatment, except it may perhaps be trephining.

Epilepsy due to syphilis is often caused by a tumor in the brain sulstance; and specific treatment will usually benefit or cure the patient.

